# Valproate activates the Snf1 kinase in *Saccharomyces cerevisiae* by decreasing the cytosolic pH

**DOI:** 10.1016/j.jbc.2021.101110

**Published:** 2021-08-21

**Authors:** Michael Salsaa, Kerestin Aziz, Pablo Lazcano, Michael W. Schmidtke, Maureen Tarsio, Maik Hüttemann, Christian A. Reynolds, Patricia M. Kane, Miriam L. Greenberg

**Affiliations:** 1 Department of Biological Sciences, Wayne State University, Detroit, Michigan, USA; 2 Department of Biochemistry and Molecular Biology, SUNY Upstate Medical University, Syracuse, New York, USA; 3 Center for Molecular Medicine and Genetics, School of Medicine, Wayne State University, Detroit, Michigan, USA; 4 Department of Emergency Medicine, School of Medicine, Wayne State University, Detroit, Michigan, USA; 5 Department of Biotechnology, University of Rijeka, Rijeka, Croatia

**Keywords:** bipolar disorder, cytosolic pH, inositol 1,4,5-triphosphate, glycolysis, mitochondrial membrane potential, Pma1 H^+^-ATPase, SNF1/AMPK, valproate, weak acid preservatives, BD, bipolar disorder, COX, cytochrome *c* oxidase, 2DG, 2-deoxyglucose, ER, endoplasmic reticulum, FBP, fructose 1,6-bisphosphate, GABA, gamma-aminobutyric acid, GAPDH, glyceraldehyde 3-phosphate dehydrogenase, IMPase, inositol monophosphatase, InsP_3_, inositol 1,4,5-triphosphate, ΔΨ_m_, mitochondrial membrane potential, PEP, phosphoenolpyruvate, PFK, phosphofructokinase, PG, phosphoglycerate, PGK, phosphoglycerate kinase, pH_c_, cytosolic pH, TMRM, tetramethylrhodamine, VPA, valproate

## Abstract

Valproate (VPA) is a widely used mood stabilizer, but its therapeutic mechanism of action is not understood. This knowledge gap hinders the development of more effective drugs with fewer side effects. Using the yeast model to elucidate the effects of VPA on cellular metabolism, we determined that the drug upregulated expression of genes normally repressed during logarithmic growth on glucose medium and increased levels of activated (phosphorylated) Snf1 kinase, the major metabolic regulator of these genes. VPA also decreased the cytosolic pH (pH_c_) and reduced glycolytic production of 2/3-phosphoglycerate. ATP levels and mitochondrial membrane potential were increased, and glucose-mediated extracellular acidification decreased in the presence of the drug, as indicated by a smaller glucose-induced shift in pH, suggesting that the major P-type proton pump Pma1 was inhibited. Interestingly, decreasing the pH_c_ by omeprazole-mediated inhibition of Pma1 led to Snf1 activation. We propose a model whereby VPA lowers the pH_c_ causing a decrease in glycolytic flux. In response, Pma1 is inhibited and Snf1 is activated, resulting in increased expression of normally repressed metabolic genes. These findings suggest a central role for pH_c_ in regulating the metabolic program of yeast cells.

Valproate (VPA) is a branched short-chain fatty acid approved by the FDA for the treatment of bipolar disorder (BD) (1, 2) and epilepsy (3, 4). Although widely used, it is not effective in all cases and can cause severe side effects, including hepatotoxicity and teratogenicity (5). Some aspects of VPA-mediated hepatotoxicity have been attributed to inhibition of mitochondrial bioenergetics by the drug (6). Long-term treatment with VPA decreases cytochrome *c* oxidase activity (COX) (7), most likely through direct inhibition (8). The therapeutic mechanism of action of VPA is not understood (9), hindering the development of more effective mood stabilizers.

Several hypotheses have been proposed to explain how mood stabilizers such as VPA and lithium mediate therapeutic effects. Because of the interrelatedness of pathways, these proposed mechanisms may not be mutually exclusive. This may be especially applicable to the inositol depletion hypothesis (10, 11) and the gamma-aminobutyric acid (GABA) potentiation hypothesis (12). Mood stabilizers such as VPA and lithium inhibit enzymes in the inositol biosynthesis pathway leading to a decrease in inositol levels (13, 14, 15, 16). Inositol depletion decreases components of the inositol 1,4,5-triphosphate (InsP_3_)/Ca^2+^ signaling pathway, which decreases Ca^2+^ release from the endoplasmic reticulum (ER). A decrease in InsP_3_/Ca^2+^ signaling may counteract the increased neuronal excitatory drive of both the excitatory and inhibitory neurons responsible for the onset of BD (10). Interestingly, inhibition of inositol monophosphatase (IMPase), a target of lithium, has been shown to stimulate the AMP-activated protein kinase (AMPK) through decreased InsP_3_ signaling, leading to a decrease in ER release and subsequent mitochondrial uptake of Ca^2+^ (17). VPA was shown to activate AMPK in mouse and human primary hepatocytes by unknown mechanisms (18). AMPK activation results in increased macroautophagy (17), a critical pathway for neuronal cell survival (19). AMPK also upregulates the transcription of PGC-1α (peroxisome proliferator-activated receptor γ coactivator 1α), the master regulator of mitochondrial biogenesis (20, 21, 22, 23). As mitochondrial dysfunction has been implicated in the pathophysiology of BD (24), increased macroautophagy and mitochondrial biogenesis through AMPK activation may contribute to the therapeutic mechanism of mood stabilizing drugs (11). In addition, AMPK-mediated phosphorylation of the GABA_B_ receptors suppresses neuronal excitation and promotes neuronal survival (25, 26). Therefore, activation of AMPK may link inositol depletion and GABA potentiation as a potential mechanism of action of mood stabilizers. Understanding how AMPK is activated by mood stabilizers may provide important insight into the therapeutic mechanism of action of the drug.

AMPK is highly conserved in eukaryotes. Snf1, the *Saccharomyces cerevisiae* homolog of AMPK and a founding member of the SNF1/AMPK family, was identified genetically in 1981, more than a decade before discovery of the mammalian homolog (27). Cross-species functionality has been demonstrated from yeast to mammalian cells, and the yeast model has been pivotal in identifying mammalian upstream activating kinases (27). Interestingly, a microarray analysis conducted to identify pathways affected by VPA in yeast revealed a pattern of altered expression of metabolic genes that is similar to the pattern observed during Snf1 activation (28, 29). In the current study, we further exploited the yeast model to gain insight into the mechanism of activation of SNF1/AMPK by VPA.

Snf1, the yeast homolog of AMPK, is activated by phosphorylation of Thr210 by one of three redundant kinases, Sak1, Elm1, and Tos3 (30). Upon activation, Snf1 regulates the activity of several transcription factors that mediate derepression of glucose-repressed genes, β-oxidation, the carnitine shuttle, the glyoxylate cycle, and gluconeogenesis (29, 31, 32). This occurs in wild-type cells when glucose is depleted during the diauxic shift (33). In addition to carbon stress, Snf1 is activated in response to other environmental stressors, even in the presence of glucose (34). Interestingly, the microarray study cited above was conducted in conditions of high glucose when Snf1 is not usually activated (28), suggesting that VPA treatment substantially impacts energy metabolism. One of the main downstream targets of Snf1 is the transcriptional repressor Mig1 (35). Upon carbon stress, Snf1 phosphorylates Mig1 leading to its nuclear export, which results in increased expression of many genes required for the use of other carbon sources, such as *SUC2* (36). While Snf1 may get activated under high-glucose conditions, it phosphorylates Mig1 only under glucose limitation. This suggests that glucose depletion regulates the Snf1-Mig1 pathway *via* two independent steps; activation of Snf1, followed by translocation of active Snf1 to the nucleus where Mig1 inactivation occurs (37).

In yeast, glycolysis is the primary metabolic pathway utilized to generate energy when glucose is present, a phenomenon known as the Crabtree effect (38). ATP produced through glycolysis serves as a critical energy source, with as much as 50% being used to fuel Pma1, the proton pump in the plasma membrane that maintains the cytosolic pH (pH_c_) (39, 40). Glycolytic activity itself is highly sensitive to pH_c_. Reductions in pH_c_ can inhibit at least two glycolytic enzymes, and this has been hypothesized to underlie the antimicrobial effects of some weak-acid food preservatives (41, 42, 43). In addition to the cellular effects of VPA discussed above, its chemistry as a weak acid predicts that it directly lowers the pH_c_, resulting in concomitant changes in energy metabolism.

Here, we report that VPA activates Snf1 even during glucose-replete conditions by a novel pH-dependent mechanism. VPA decreased the pH_c_ and inhibited proton efflux, consistent with decreased activity of the plasma membrane proton pump Pma1. Snf1 activation could be recapitulated by direct inhibition of Pma1 with omeprazole, which decreases the pH_c_. These findings support a novel mechanism of Snf1 activation by VPA mediated through decreasing the pH_c_.

## Results

### VPA activates Snf1 independent of inositol depletion and cytochrome *c* oxidase inhibition

**Figure 1:**
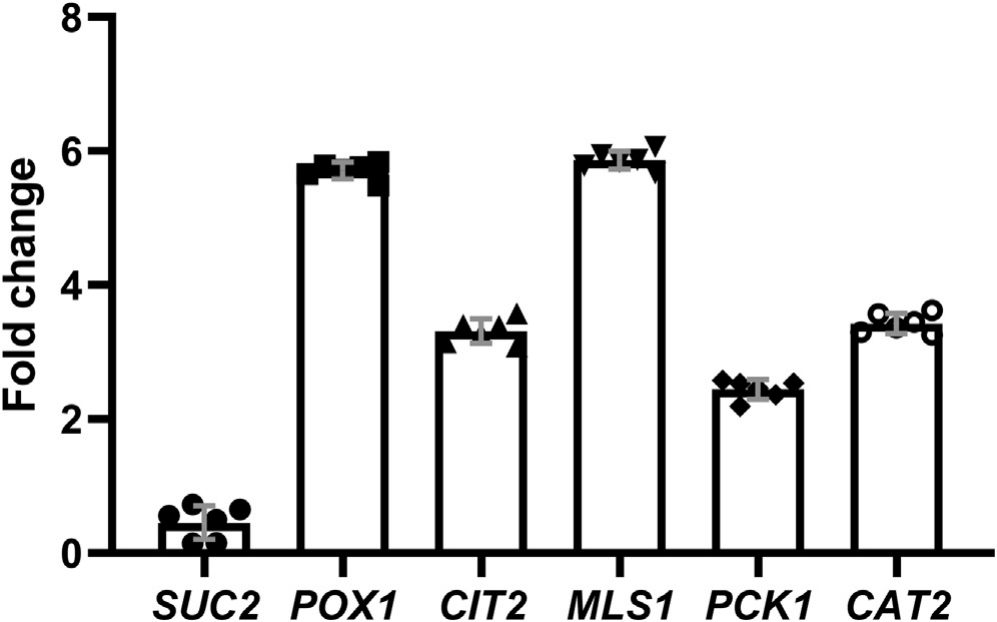
**qPCR analysis of Snf1-regulated genes**. Cells grown in I− medium to the mid-log phase were treated with VPA (1 mM) for 30 min. Total RNA was extracted and mRNA levels of Snf1-regulated genes were determined by real-time PCR. Expression was normalized to mRNA levels of the internal control, *ACT1*. Values are mean ± SD from three independent experiments with technical duplicates.

**Figure 2:**
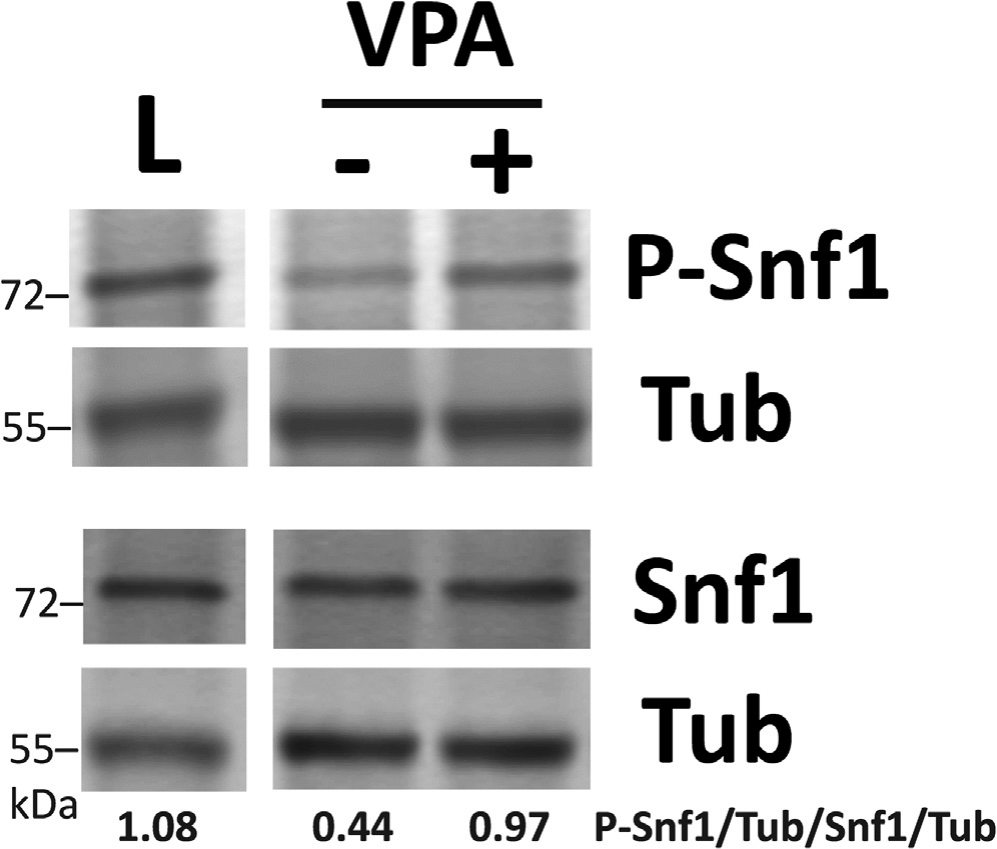
**VPA activates Snf1**. Cells cultured in I− medium containing high glucose (2%) until the mid-log phase were either treated with or without VPA (1 mM) for 30 min or switched to low glucose (0.05%) for 1 h (L). Parallel samples were loaded on two separate gels and levels of phosphorylated and endogenous Snf1 were determined by Western blot analysis. Both membranes were incubated with anti-tubulin antibody (Tub) to normalize for protein loading. Values indicate the relative intensity of phosphorylated Snf1 normalized to tubulin divided by that of total Snf1 protein normalized to tubulin. The figure is representative of three biological replicates.

A previously reported microarray analysis of the yeast gene expression response to VPA suggested altered expression of metabolic genes (28). qPCR analysis indicated no change in *SUC2* (invertase; sucrose metabolism) expression and increased expression of *POX1* (fatty-acyl CoA oxidase; β-oxidation), *CIT2* and *MLS1* (citrate synthase 2 and malate synthase; glyoxylate cycle), *PCK1* (phosphoenolpyruvate carboxykinase; gluconeogenesis), and *CAT2* (carnitine acetyl-CoA transferase; carnitine shuttle) in response to VPA treatment (1 mM, 30 min) (Fig. 1). This gene expression profile is similar to the pattern observed following Snf1 activation (29, 31, 32) and is specific for Snf1 activation under glucose replete conditions, which do not result in Mig1 phosphorylation and, as a result, increased *SUC2* expression (37). Therefore, we assayed Snf1 activation by determining levels of Thr210-phosphorylated Snf1 (44). Western blot analysis revealed that VPA increased levels of activated (phosphorylated) Snf1 (Fig. 2).

**Figure 3:**
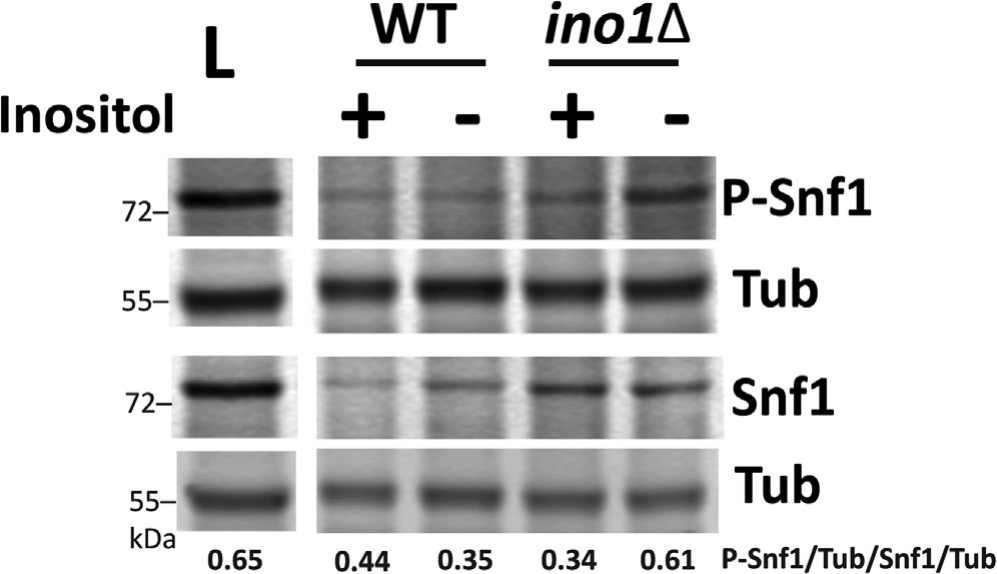
**Inositol depletion activates Snf1**. WT and *ino1*Δ cells were cultured in I+ medium until the mid-log phase. Cells were collected on a filter, washed twice with dH_2_O, and transferred to fresh medium with (I+) or without inositol (I−) for 4 h or switched to low glucose (0.05%) I− medium for 1 h (L). Parallel samples were loaded on two separate gels. Both membranes were incubated with anti-tubulin antibody (Tub) to normalize for protein loading. Values indicate the relative intensity of phosphorylated Snf1 normalized to tubulin divided by that of total Snf1 protein normalized to tubulin. The figure is representative of three biological replicates.

**Figure 4:**
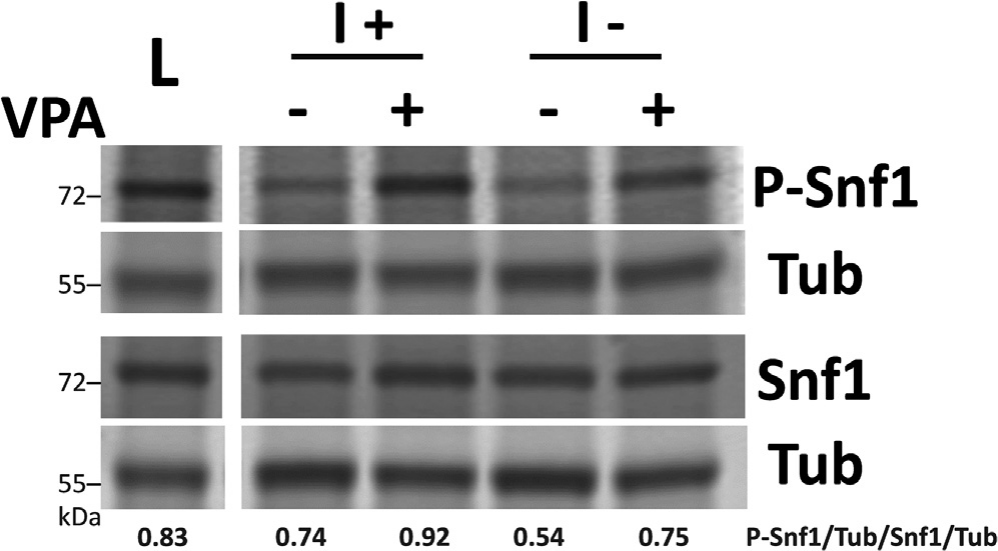
**VPA activates Snf1 in the presence of inositol**. WT cells were cultured in the presence or absence of inositol until mid-log phase and treated with VPA (1 mM) for 4 h or switched to low glucose (0.05%) for 1 h (L). Parallel samples were loaded on two separate gels. Both membranes were incubated with anti-tubulin antibody (Tub) to normalize for protein loading. Values indicate the relative intensity of phosphorylated Snf1 normalized to tubulin divided by that of total Snf1 protein normalized to tubulin. The figure is representative of three biological replicates.

Previous studies have shown that VPA causes inositol depletion in yeast (45) and mammalian brain tissue (16). Inhibition of IMPase, which decreases inositol levels (14), activates AMPK in several mammalian cell lines (17). To explore the possibility that inositol depletion activates Snf1, we utilized the *ino1*Δ mutant, which cannot synthesize inositol and undergoes “inositol-less death” after two doublings (46). Shifting *ino1*Δ mutant cells to medium without inositol (I−) for 4 h increased Snf1 phosphorylation (Fig. 3). Snf1 phosphorylation was not increased in wild-type cells, which can synthesize inositol in I− medium. These findings indicate that inositol depletion activates Snf1. To determine if the mechanism of Snf1 activation by VPA is mediated by decreased inositol levels, wild-type cells were treated with VPA in the presence or absence of inositol for 4 h. VPA increased Snf1 phosphorylation in the presence or absence of inositol (Fig. 4), demonstrating that activation of Snf1 by VPA is independent of inositol levels.

VPA decreases mitochondrial bioenergetics in hepatocytes (6). More recently, we reported that VPA directly inhibits cytochrome *c* oxidase (COX) (8), the last and rate limiting enzyme of the electron transport chain. Inhibition of COX decreases mitochondrial bioenergetics and ATP levels and activates AMPK (47). To test if VPA activates Snf1 through inhibition of COX, cells were treated with the COX inhibitor potassium cyanide (0.2 mM) for 30 min. Unlike VPA, potassium cyanide did not activate Snf1 when cells were grown in high glucose (data not shown). This is consistent with a previous finding that antimycin A, a specific inhibitor of complex III, did not activate Snf1 when cells were grown in high glucose (34). Taken together, these results indicate that VPA activates Snf1 through a mechanism independent of inositol or COX inhibition.

### VPA reduces proton efflux and lowers pH_c_

**Figure 5:**
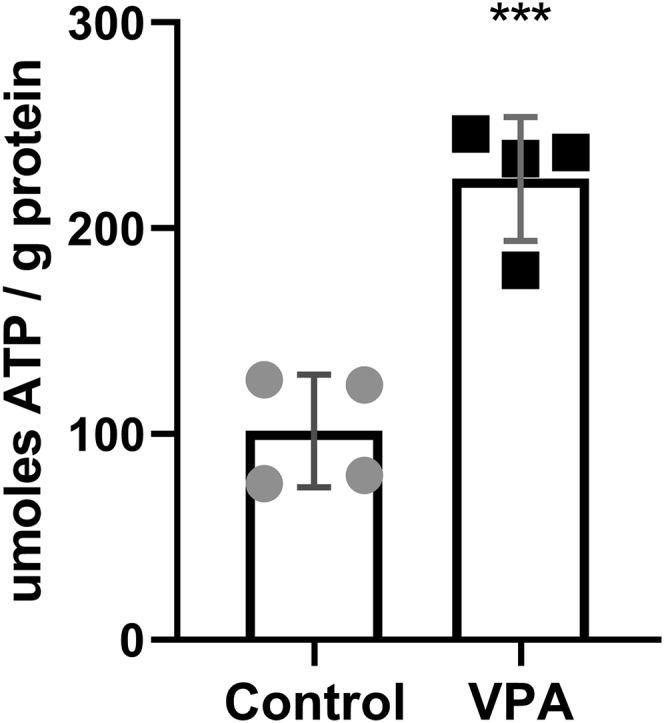
**VPA increases ATP levels**. Cells were cultured until the mid-log phase, treated with VPA (0.6 mM) for 30 min, and ATP levels were determined as described under Experimental procedures. Data shown are mean ± SD (n = 4). Unpaired Student's *t* test was used for statistical analysis (∗∗∗*p* < 0.001).

The primary function of SNF1/AMPK is to conserve cellular energy and increase ATP levels (48). In mammalian cells, AMPK is directly activated by AMP when the AMP/ATP ratio increases (49). Unlike AMPK, Snf1 is not directly activated by AMP, but it has been suggested that Snf1 may be activated by elevated ADP levels (50). Therefore, we addressed the possibility that VPA treatment activates Snf1 as a result of increased ATP hydrolysis. Surprisingly, we observed that VPA treatment resulted in an approximate doubling of ATP levels after 30 min (Fig. 5).

**Figure 6:**
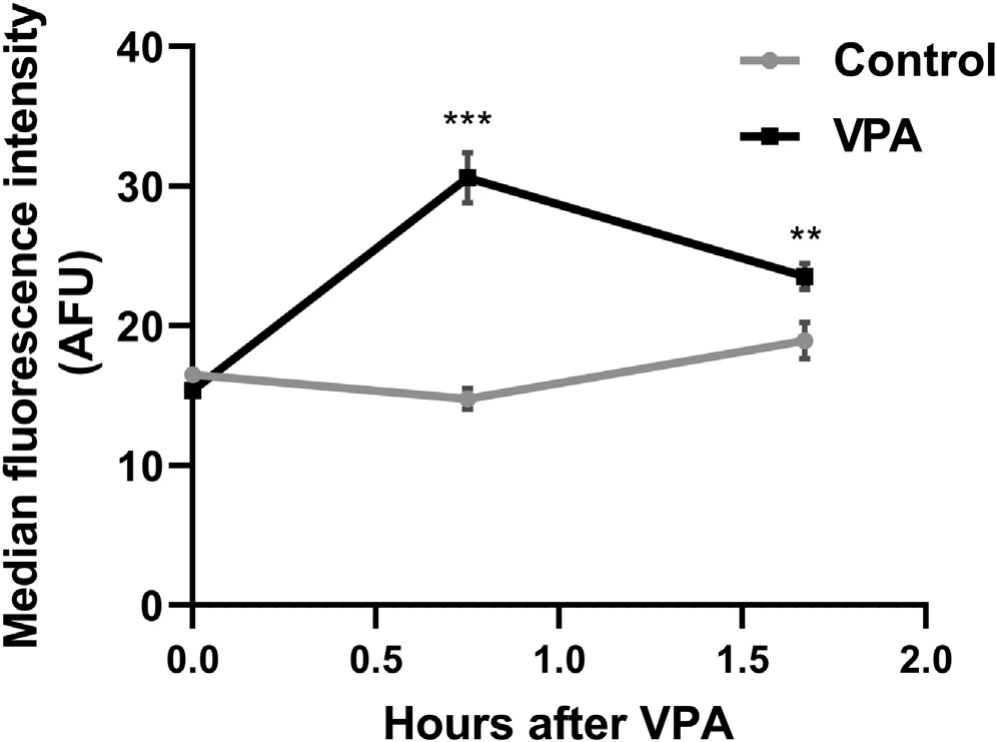
**VPA increases ΔΨ**_**m**_. Cells were cultured until the mid-log phase and treated with VPA (1 mM). TMRM (500 nM) was freshly added to separate batches of the same culture 30 min before each sample was collected. ΔΨ_m_ was determined by measuring TMRM fluorescence using flow cytometry. Data presented are in arbitrary fluorescence units (AFUs). Data shown are mean ± SD (n = 5). Unpaired Student's *t* test was used for statistical analysis (∗∗∗*p* < 0.001).

**Figure 7:**
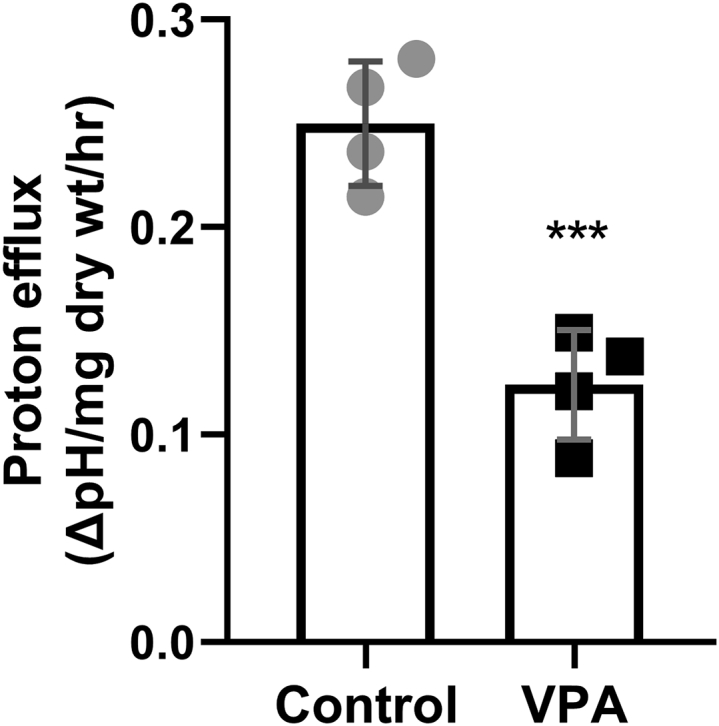
**VPA decreases proton efflux.** Cells were cultured until the mid-log phase, washed, and stored on ice to starve for glucose. Cells were then resuspended in a sorbitol solution and a stable pH reading was achieved. After initiating proton efflux by glucose addition, the suspension was treated with dH_2_O (control) or 1 mM VPA and the decrease in pH was followed for 15 min. The proton efflux rate is expressed as the difference between the pH reading before and after the treatment, normalized to the dry weight of the cells, and divided by treatment time. Data shown are mean ± SD (n = 4). Unpaired Student's *t* test was used for statistical analysis (∗∗∗*p* < 0.001).

A possible explanation for increased ATP levels is that VPA inhibits cellular processes that consume ATP. The plasma membrane H^+^-ATPase pump, Pma1, is a major consumer of cellular ATP in yeast, where it functions to maintain the cytosolic pH by actively pumping H^+^ out of the cell (51, 52). Pharmacologic inhibition of Pma1 leads to a 2-fold increase in cellular ATP levels (39). This increase in ATP activates the mitochondrial F_0_F_1_-ATPase (complex V) in the reverse direction, leading to increased hydrolysis of excess ATP and pumping of H^+^ out of the mitochondrial matrix (40). Accumulation of protons in the intermembrane space increases the mitochondrial membrane potential (ΔΨ_m_) (40). Similar to the effect of Pma1 inhibition, VPA treatment led to an increase in ΔΨ_m_ (Fig. 6). Increased ATP levels and ΔΨ_m_ after VPA treatment suggested that VPA inhibits Pma1. The proton pumping activity of Pma1 results in rapid extracellular acidification (*i.e.*, proton efflux) in the presence of glucose (53), which is used as an indicator of Pma1 activity (41, 54, 55). Proton efflux in control and VPA-treated cell cultures was assayed by determining the rate of change in extracellular pH normalized to the dry weight of cells (41, 56). VPA decreased the acidification rate, as indicated by a smaller glucose-induced shift in pH (Fig. 7), suggesting that VPA directly or indirectly inhibits Pma1 activity. To test if VPA directly inhibits Pma1, isolated plasma membranes were preincubated with 1 mM VPA on ice for 30 min and Pma1 activity was measured using the coupled enzyme assay (57). VPA-treated samples had 91.1 ± 2.9% the activity of the buffer-treated samples, suggesting there was little, if any, inhibition. Direct addition of VPA along with the membranes to the ATPase assay (without preincubation) also gave very little inhibition. The observed reduction in activity was similar to that obtained in assays of VPA with vacuolar vesicles (which do not contain Pma1), suggesting that it represents an effect on the coupled enzyme assay rather than specific inhibition of ATPase activity from the membrane preparations. These results indicate that VPA inhibition of Pma1 is indirect. One potential mechanism of modulating Pma1 activity is through phosphorylation, as is the case in response to glucose (58). Cells were incubated with VPA (1 mM) for 30 min and plasma membranes were isolated. VPA did not affect Pma1 specific activity (data not shown), indicating that Pma1 inhibition by VPA is not mediated by posttranslational modification. Another mechanism of regulating Pma1 activity is *via* modulation of Pma1 plasma membrane levels through endocytosis or protein sorting (59, 60). Pma1 levels at the plasma membrane were determined through indirect immunofluorescence (60). Incubation of cells with VPA (1 mM) for 30 min did not decrease Pma1 levels at the plasma membrane (data not shown).

**Figure 8:**
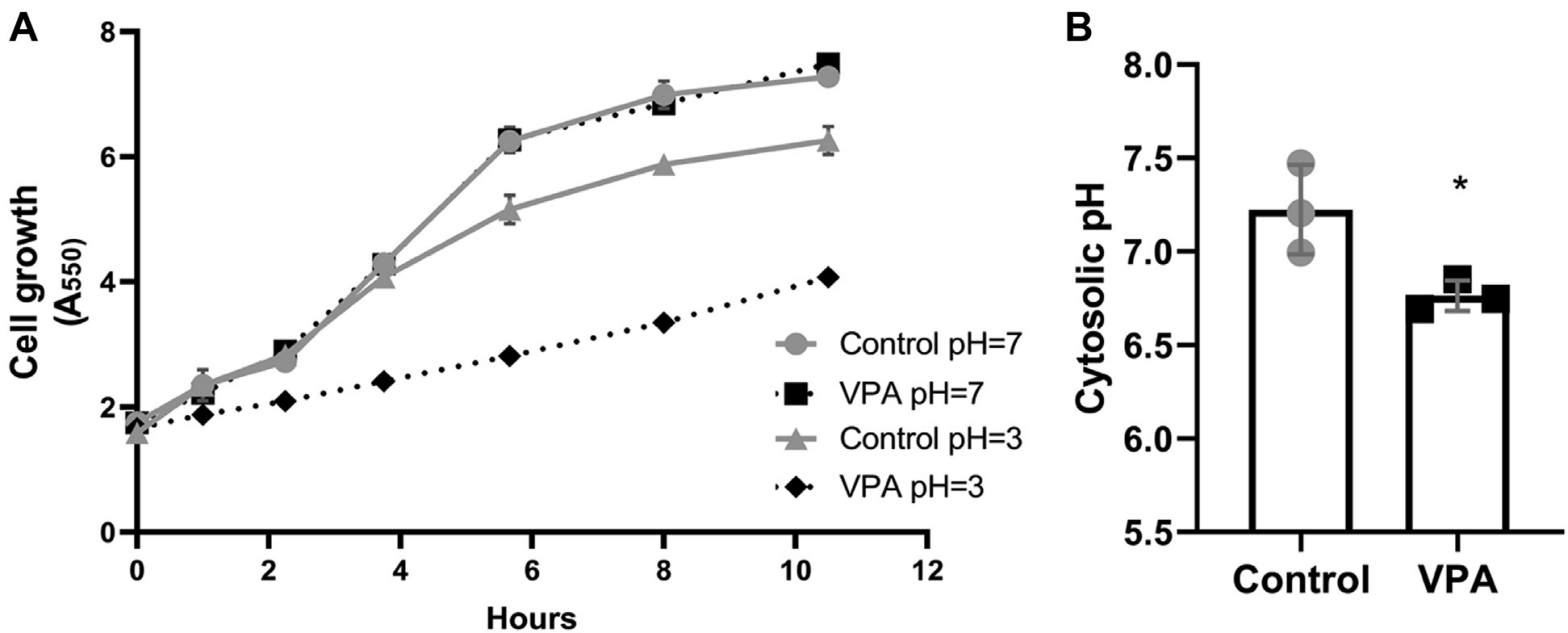
**VPA exhibits weak acid properties.***A*, effect of VPA on yeast growth at extracellular pH 7.0 or 3.0. Cells cultured to the mid-log phase were split equally into four tubes and washed once with fresh medium adjusted to either pH 7.0 or 3.0. Cells were resuspended in similarly pH-adjusted fresh medium and either VPA (1 mM) or dH_2_O was added to the tubes at time zero. Growth was monitored by absorbance at 550 nm. The medium of cells grown at pH 7.0 was continually readjusted to pH 7.0 with 1 M NaOH. Data shown are mean ± SD (n = 3). *B*, response of pH_c_ to VPA. WT cells transformed with a plasmid expressing pHluorin under control of a yeast promoter were grown to the mid-log phase, washed once in 1 mM MES containing 2% glucose, and resuspended in the same buffer. VPA (1 mM) was added for 30 min and fluorescence intensity was measured at excitation wavelengths 405 and 485 nm and emission wavelength 508 nm. pH values were determined from fluorescence ratios as described under Experimental procedures. Data shown are mean ± SD (n = 3). Unpaired Student's *t* test was used for statistical analysis (∗*p* < 0.05).

Weak acids, including short-chain fatty acids, are hypothesized to directly decrease the pH_c_ in yeast, as they are able to traverse the plasma membrane in the protonated state at low extracellular pH and release their protons in the neutral cytoplasm (61, 62, 63). VPA decreases yeast growth when added to cells in the mid-log phase (64). Similarly, VPA (1 mM) decreased growth when added to fresh medium adjusted to pH 3.0 (Fig. 8*A*). However, when the medium pH was continually readjusted to 7.0, VPA did not affect growth (Fig. 8*A*). We therefore tested the possibility that VPA decreases the pH_c_, utilizing a pH-sensitive variant of green fluorescent protein, pHluorin, developed to provide real-time ratiometric measurements of the pH_c_ in yeast (65, 66). Cells were administered VPA and the pH_c_ of pHluorin-expressing cells was assayed *in vivo* by measuring fluorescence intensity at two wavelengths (67). VPA (1 mM) decreased the pH_c_ from 7.2 to 6.8 (Fig. 8*B*).

**Figure 9:**
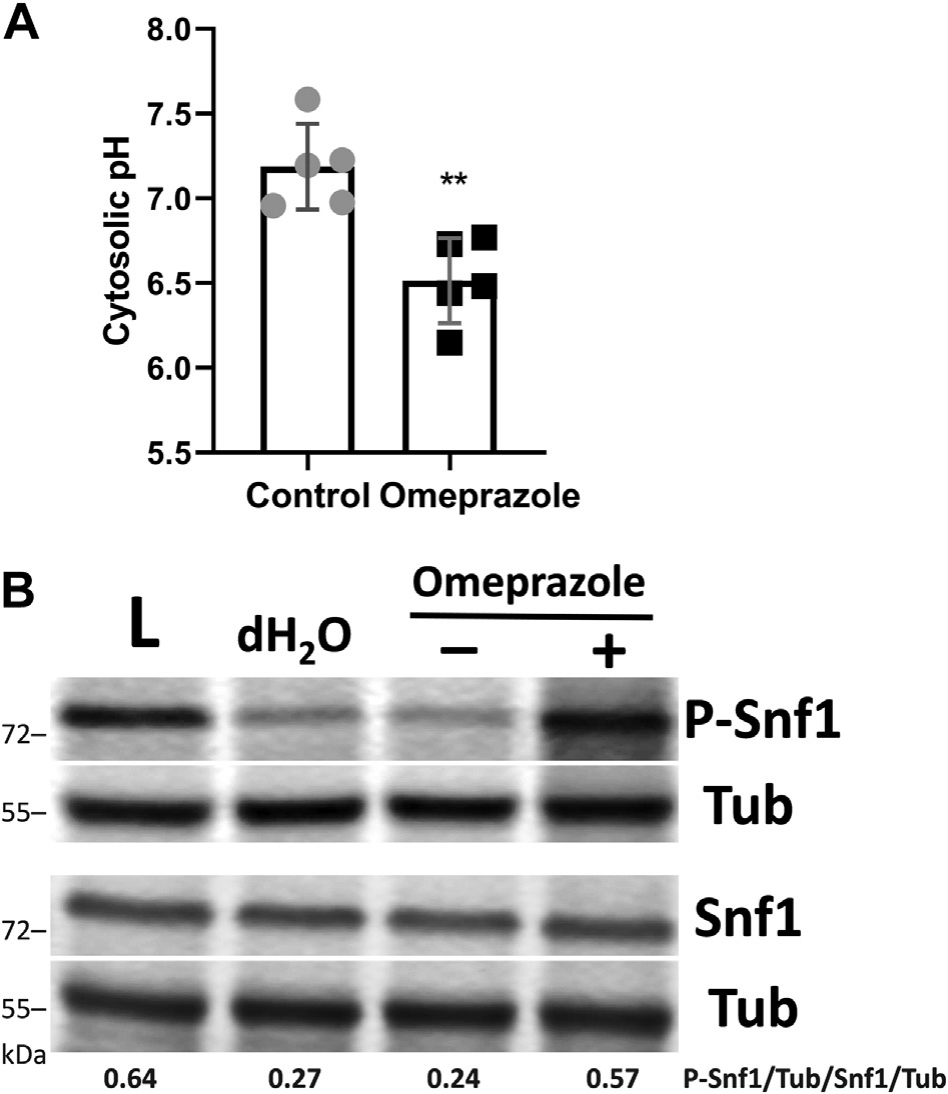
**The Pma1 inhibitor omeprazole decreases the cytosolic pH and activates Snf1.** Cells were cultured in I− medium until the mid-log phase, then incubated with acid-activated omeprazole (200 μM) or control (DMSO) for 30 min. *A*, response of pH_c_ to omeprazole. WT cells transformed with a plasmid expressing pHluorin under control of a yeast promoter were grown to the mid-log phase, washed twice in 1 mM MES containing 2% glucose, and resuspended in the same buffer. Fluorescence intensity was measured at excitation wavelengths 405 and 485 nm and emission wavelength 508 nm. pH values were determined from fluorescence ratios as described under Experimental procedures. Data shown are mean ± SD (n = 5). Unpaired Student's *t* test was used for statistical analysis (∗∗*p* < 0.01). *B*, Western blot of P-Snf1 response to omeprazole. Cells were treated with omeprazole (+), DMSO (−), or dH_2_O for 30 min or switched to low glucose (0.05%) for 1 h (L). Parallel samples were loaded on two separate gels. Both membranes were incubated with anti-tubulin antibody (Tub) to normalize for protein loading. Values indicate the relative intensity of phosphorylated Snf1 normalized to tubulin divided by that of total Snf1 protein normalized to tubulin. The figure is representative of three biological replicates.

Using an independent approach to determine if decreasing the pH_c_ could activate Snf1, we determined the effect on Snf1 phosphorylation of the proton pump inhibitor omeprazole, which has been shown to inhibit Pma1 activity (68). Consistent with this effect, we observed a decrease in the pH_c_ in omeprazole-treated yeast cells (Fig. 9*A*) (40). As shown in Figure 9*B*, omeprazole treatment (200 μM, 30 min) resulted in a somewhat higher degree of phosphorylation of Snf1 than that observed in cells treated with 1 mM VPA (Fig. 2). This could reflect the greater decrease in pH_c_ observed in the presence of omeprazole (Fig. 9*A*) than in the presence of VPA (Fig. 8*B*) at the concentrations used. Taken together, these findings support a model in which decreased pH_c_ serves as a signal for Snf1 activation.

### VPA decreases carbon flux through the pH-sensitive GAPDH/PGK step of glycolysis

**Figure 10:**
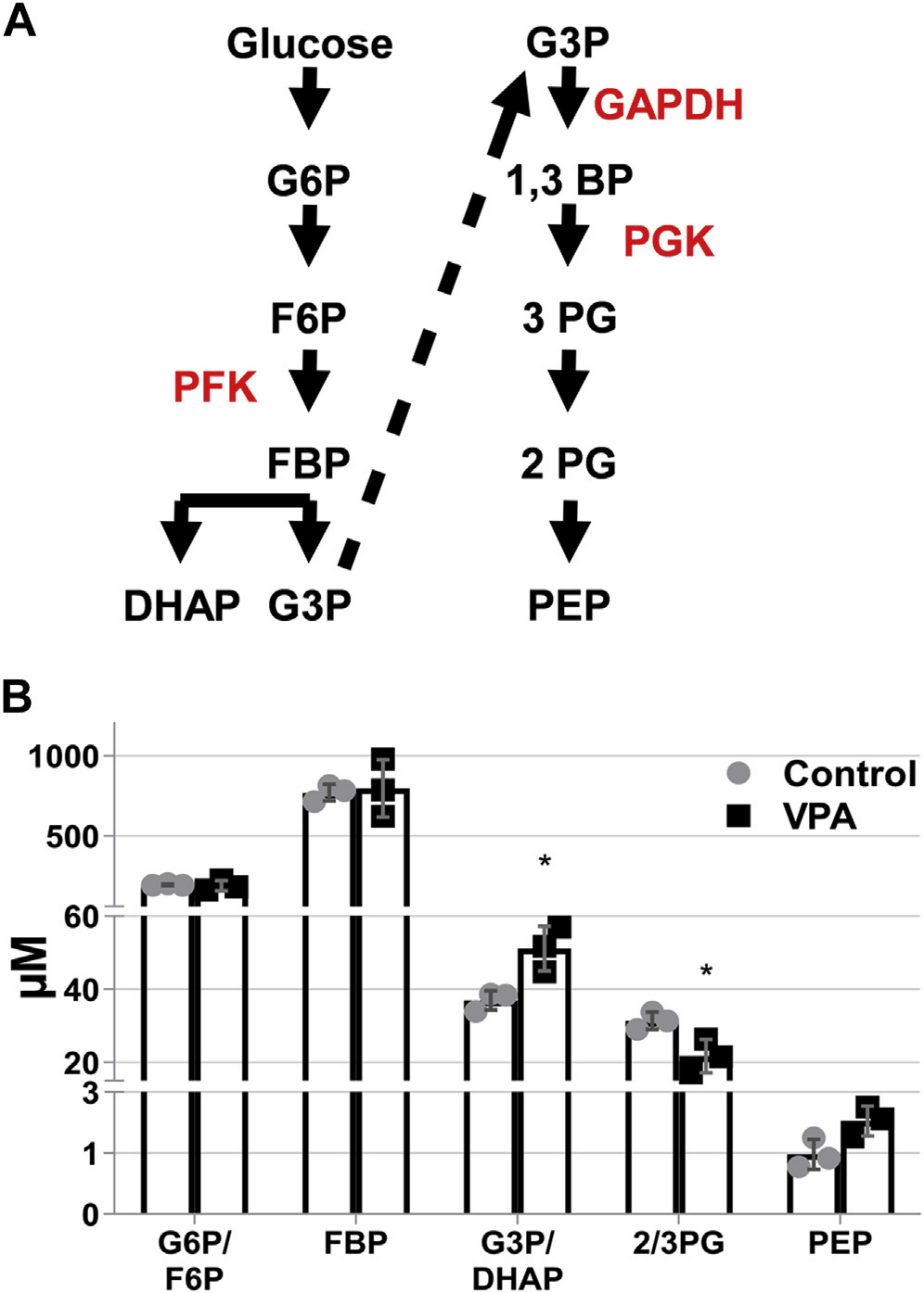
**VPA decreases 2/3 phosphoglycerate levels.** Cells were cultured until the mid-log phase and treated with VPA (1 mM) for 30 min, lysed, and glycolytic metabolites were determined by mass spectrometry. The values represent the concentration of each metabolite (μM) normalized to cell density measured at A_550_. Data shown are mean ± SD (n = 3). Unpaired Student's *t* test was used for statistical analysis (∗*p* < 0.05). *A*, glycolytic pathway with pH-sensitive enzymes in *red*. *B*, levels of glycolytic intermediates in response to VPA. DHAP, dihydroxyacetone phosphate; F6P, fructose 6-phosphate; FBP, fructose 1,6-bisphosphate; GAPDH, glyceraldehyde 3-phosphate dehydrogenase; G3P, glyceraldehyde 3-phosphate; G6P, glucose 6-phosphate; PEP, phosphoenolpyruvate; PFK, phosphofructokinase; PG, phosphoglycerate; PGK, phosphoglycerate kinase.

The glycolytic reactions catalyzed by phosphofructokinase (PFK) and glyceraldehyde 3-phosphate dehydrogenase/phosphoglycerate kinase (GAPDH/PGK) (Fig. 10*A*) are pH-sensitive, as their activities are increased with increased pH and inhibited with decreased pH (42, 69). PFK is the key control point of glycolytic regulation by pH and is inhibited in yeast by treatment with the weak acid benzoate (10 mM), yet glycolytic activity is reduced by low pH even when the PFK step is circumvented by providing fructose 1,6-bisphosphate (FBP) as a glycolytic substrate (42, 43). The latter observation has been attributed to pH-dependent inhibition of GAPDH and/or PGK (which are not readily distinguishable) (42). Therefore, a VPA-mediated decrease in pH_c_ would be expected to decrease glycolysis at these steps. Consistent with this possibility, cells treated with VPA exhibited a 30% increase in glyceraldehyde 3-phosphate/dihydroxyacetone phosphate levels and a 30% decrease in 2/3-phosphoglycerate (Fig. 10*B*), suggesting that flux of carbon through GAPDH/PGK was decreased by VPA. As 1,3-bisphosphoglycerate, the product of GAPDH, is not a commonly used standard in mass spectrometry analysis, we were not able to distinguish which of these enzymes (GAPDH or PGK) was affected by the treatment. The levels of FBP were not affected by VPA (Fig. 10*B*), suggesting that the decrease in pH_c_ by 1 mM VPA may not be large enough to affect the activity of PFK (43). This suggests that GAPDH/PGK activity is more sensitive to a small decrease in neutral pH than PFK, which is consistent with a report in which GAPDH activity was reduced at pH 7.0 to the same extent as PFK at pH 6.6 (69).

Ullah and colleagues reported that yeast can restore the decrease in pH_c_ by weak acids, which would remove the inhibition on GAPDH/PGK (70). In a previous study, VPA inhibited mitochondrial bioenergetics and increased glycolytic activity after 5 h (8). Therefore, it is likely that cells respond to the decrease in GAPDH/PGK and mitochondrial activity by increasing glycolytic gene expression, resulting in the observed increase in glycolysis (8).

Collectively, these findings suggest that VPA lowers the pH_c_, causing a decrease in the glycolytic conversion of glyceraldehyde 3-phosphate/dihydroxyacetone phosphate to 2/3-phosphoglycerate, signaling the downstream activation of Snf1 and inhibition of Pma1.

## Discussion

**Figure 11:**
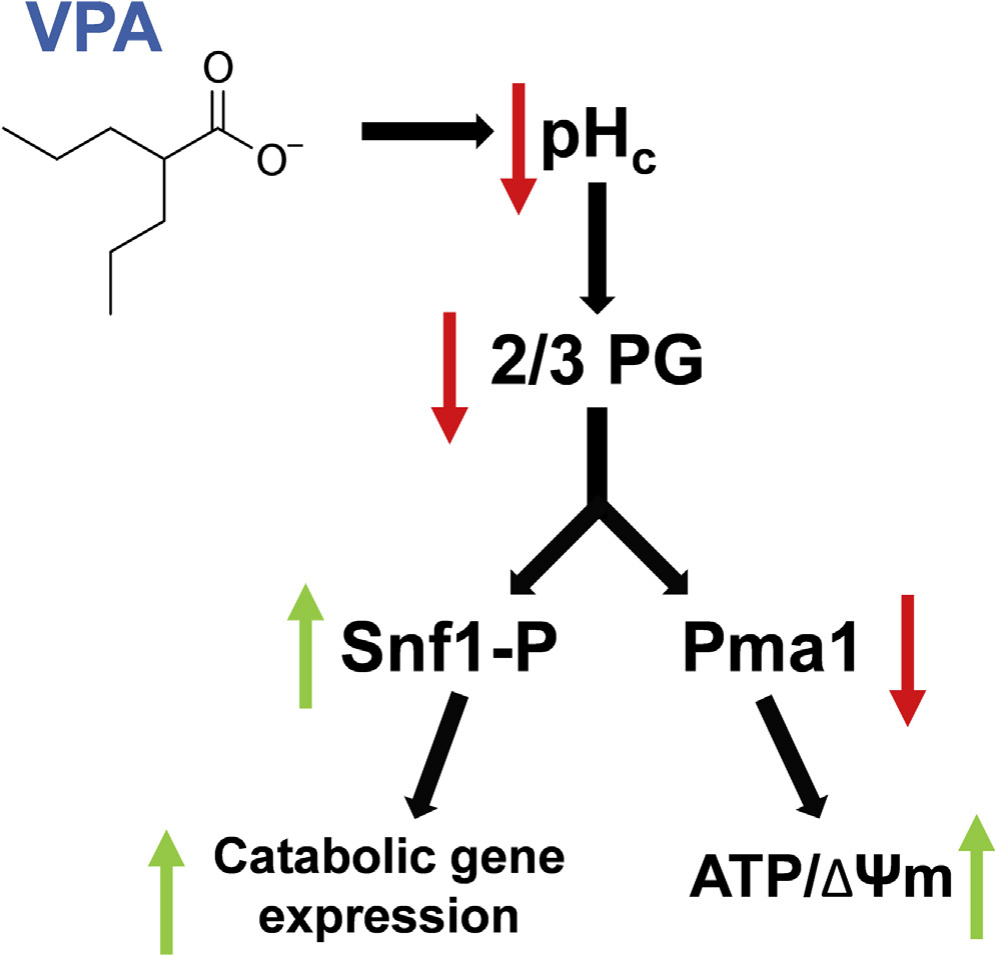
**Model: mechanism of Snf1 activation by VPA.** VPA acts as a weak acid to decrease the pH_c_. A reduction in pH_c_ inhibits glycolytic flux through the GAPDH/PGK step of glycolysis, leading to a decrease in the metabolite 2/3 PG. This alteration in glycolytic carbon flow leads to activation of Snf1 and inhibition of Pma1 activity. Activation of Snf1 switches metabolism to catabolism while inhibition of Pma1 conserves cellular ATP. These changes in yeast physiology and metabolism facilitate the transition to the stationary phase of growth.

The current study indicates that acute treatment with VPA activates the Snf1 kinase and increases catabolic gene expression. Activation of Snf1 by VPA is independent of decreasing inositol levels and inhibiting mitochondrial COX, previously established effects of VPA. Instead, we found that VPA decreased the pH_c_ to an extent that was sufficient to reduce carbon flux through the pH-sensitive glycolytic step catalyzed by GAPDH/PGK. This results in two important outcomes: (1) activation of Snf1, switching cellular metabolism to catabolism, and (2) inhibition of the plasma membrane proton pump, Pma1, likely in order to conserve cellular ATP levels. This model is summarized in Figure 11.

The current study is also the first to report increased Snf1 phosphorylation in response to inositol depletion. Although activation of AMPK by inositol depletion has yet to be tested directly, it has been previously shown that inhibition of IMPase, which decreases inositol levels (14), can activate AMPK in mammalian cells (17). Therefore, decreasing inositol levels may still be part of the mechanism of AMPK activation by VPA in mammalian cells (18). From our study, we cannot rule out the possibility that decreasing inositol levels contributes to Snf1 activation by VPA in the absence of exogenous inositol. However, because VPA activates Snf1 in the presence of abundant inositol, it is likely that Snf1 activation by VPA is mainly mediated by a different mechanism.

Previous studies have established the ability of weak acid preservatives to lower the pH_c_ and attributed the mechanism to the classical weak acid theory (61, 63). Weak acids exist in equilibrium between protonated and deprotonated states. At low extracellular pH, a weak acid is mostly in the protonated form, which can diffuse through the plasma membrane into the cytosol. In support, VPA inhibited yeast growth at a medium pH of 3.0 but did not inhibit growth when the extracellular pH was continually readjusted to 7.0 (Fig. 8*A*), which indicates that VPA enters yeast cells through passive diffusion in the protonated form. In a neutral cytosolic environment, most of the weak acid dissociates, leading to the release of a proton and an anion (62). The accumulation of released protons and anions decreases the pH_c_ and initiates an energetically demanding cycle of preservative anion pumping, which has been hypothesized as part of the mechanism of growth inhibition by weak acid food preservatives (62). As a weak acid, VPA likely has a similar effect on cells. In the current study, VPA was added during the mid-log growth phase when the extracellular pH is low (pH 3.1, data not shown), whereas the pH_c_ is maintained around neutral (71). Indeed, 1 mM VPA led to a 0.4–0.5 unit decrease in the pH_c_ (Fig. 8*B*).

Stratford and coworkers proposed the alternative hypothesis that the mechanism whereby valeric and sorbic acids decrease the pH_c_ is through direct inhibition of Pma1, possibly by altering membrane fluidity (41). Specific pharmacological inhibition of Pma1 H^+^ pumping decreases the pH_c_ (Fig. 9*A*) (40). VPA caused an increase in ATP and ΔΨ_m_, as well as a decrease in proton efflux, indicators of Pma1 inhibition. However, preincubation of isolated plasma membranes with VPA did not affect the ATPase activity of Pma1, suggesting that VPA indirectly inhibits the pump. Additionally, incubation of cells with VPA for 30 min did not affect Pma1 ATPase activity in isolated plasma membranes or Pma1 levels at the plasma membrane (data not shown). Taken together, these results indicate that VPA indirectly inhibits Pma1, although not by mechanisms we tested.

What causes indirect inhibition of the pump? We suggest that perturbation of glycolysis is the cause of inhibition of Pma1 by VPA. In support, inhibition of glycolysis with the glucose analog 2-deoxyglucose (2DG), which cannot be metabolized past the first glycolytic step, decreases glucose-induced extracellular acidification (72). In addition, perturbation of glycolysis is likely the signal for activation of Snf1 as 2DG treatment increases Snf1 phosphorylation in high glucose (73). In summary, our data support a model in which VPA, as a weak acid, decreases the pH_c_. A decrease in the pH_c_ causes inhibition of a glycolytic step, which serves as an underlying signal for both Snf1 activation and inhibition of Pma1 (Fig. 11).

A decrease of pH_c_ in response to VPA can explain previous therapeutically relevant observations in yeast. VPA decreases *INO1* expression, which encodes the first and rate-limiting enzyme of inositol biosynthesis, during the first hour (64). The decrease in *INO1* is likely mediated by translocation of the transcriptional repressor Opi1 from the ER, where it binds phosphatidic acid, to the nucleus (74). This can occur in response to a small decrease in pH_c_, which changes the protonation state of phosphatidic acid and results in a loss of binding with Opi1 (75). In addition, VPA indirectly inhibits the enzyme encoded by *INO1*, *myo*-inositol phosphate synthase, which results in deceased inositol levels (76). While inhibition of *myo*-inositol phosphate synthase by VPA has not been attributed to a decrease in pH_c_, other short-chain carboxylic acids can also inhibit the enzyme (77). The inositol depletion hypothesis is one of the main hypotheses to explain the therapeutic mechanism of action of VPA through decreased InsP_3_/Ca^2+^ signaling (10). Inositol depletion can also activate Snf1 (Fig. 3) and possibly AMPK (17), which can suppress neuronal excitation through phosphorylation of GABA_B_ receptors (26).

The current study establishes an important and novel link between the Snf1 kinase, pH_c_, and yeast metabolism. We propose that the pH_c_ serves as a regulatory growth signal for yeast through its effect on glycolytic flux and the master metabolic regulator Snf1. There is a strong body of research suggesting that the pH_c_ serves as a signaling mechanism linking nutrient availability, especially glucose, with growth physiology in yeast (78, 79, 80). Orij and colleagues have demonstrated that pH_c_ is regulated by glucose levels and that a drop in pH_c_ resulting from glucose starvation coincides with growth arrest (71). Importantly, pH_c_ appears to unidirectionally dictate growth rate, as growth can be precisely tuned by experimentally altering pH_c_ (81). Signaling through the pH_c_ has the advantages of being both rapid and widespread. This means that parameters such as enzyme activity and protein binding/localization can be regulated in multiple downstream pathways simultaneously (62, 75). The exact mechanism underlying growth regulation by pH_c_ has remained obscure. The current study contributes novel insights into this mechanism by invoking the master metabolic regulator Snf1. Specifically, our study provides the first evidence of Snf1 being activated by decreased pH_c_. As mentioned above, previous studies have shown Snf1 activation as a result of glucose depletion. Our findings suggest that activation of Snf1 in this context depends more precisely on glycolytic flux rather than absolute glucose levels. In this way, decreased pH_c_ acts as a signaling mechanism to reduce glycolytic flux, activate Snf1, and slow yeast growth, corresponding to the stationary phase of yeast growth.

## Experimental procedures

### Yeast strains, growth medium, and conditions

The *S. cerevisiae* strains used in this study were SF838-5Aα, BY4741 *MATa* (WT), and the BY4741-derived mutant *ino1*Δ (ino1Δ::KanMX4) purchased from Invitrogen. All experiments utilized the BY4741 yeast background, except for determination of Pma1 ATPase activity, which utilized the SF838-5Aα background. Cells were maintained on YPD medium (2% bactopeptone, 1% yeast extract, 2% glucose) and grown in synthetic minimal medium without inositol (I−), which contained all the essential components of Difco yeast nitrogen base (except inositol), 2% glucose, 0.2% ammonium sulfate, vitamin mix, histidine (20 mg/L), methionine (20 mg/L), leucine (60 mg/L), and uracil (40 mg/L). Where indicated, inositol was added at a concentration of 75 μM (I+). VPA (sodium valproate, Sigma) was added to a final concentration of either 0.6 mM or 1 mM while controls were administered dH_2_O. Absorbance was measured at 550 nm to monitor growth in liquid cultures. All incubations were at 30 °C.

### VPA and omeprazole treatment

Cells were precultured overnight in synthetic minimal medium, harvested, washed twice, and resuspended in fresh medium. Cells were inoculated to a final A_550_ of 0.05 and cultured to the mid-log phase (A_550_ = 0.5–0.7). VPA or dH_2_O was then added and cultures were incubated for the indicated time. For omeprazole experiments, omeprazole (200 μM; Sigma) was dissolved in DMSO and acid-activated by adding 0.1 N HCl for 30 min before treatment. Controls were administered acidified DMSO.

### Effect of VPA on growth at extracellular pH 7.0 or 3.0

Cells cultured to the mid-log phase were split equally into four tubes, centrifuged, and washed once with fresh medium adjusted to either pH 7.0 or 3.0. Cells were resuspended in similarly pH-adjusted fresh medium and either VPA (1 mM) or dH_2_O was added to the tubes. The pH of culture medium for cells grown in pH 7.0 was continually monitored and the pH was readjusted to 7.0 with 1 M NaOH as needed.

### Quantitative PCR (qPCR)

Cells were harvested, and total RNA was isolated using the RNeasy Plus Mini kit from Qiagen. Complementary DNA (cDNA) was synthesized using a Transcriptor First Strand cDNA Synthesis Kit (Roche Applied Science) according to the manufacturer's recommendations. qPCRs were performed in a 20 μl volume using Brilliant III Ultra-Fast SYBR Green qPCR Master Mix (Agilent Technologies). The primers for qPCR are listed in Table 1. PCRs were initiated at 95 °C for 10 min for denaturation followed by 40 cycles consisting of 30 s at 95 °C and 60 s at 55 °C. RNA levels were normalized to the *ACT1* gene. Primer sets were validated according to the Methods and Applications Guide from Agilent Technologies (Table 1). Optimal primer concentrations were determined, and primer specificity of a single product was monitored by a melt curve following the amplification reaction. All primers were validated by measurement of PCR efficiency and have calculated reaction efficiencies between 90 and 110%.

**Table 1:** Primers used for qPCR analyses

Gene	Primer	Sequence
*ACT1*	Forward	GATTCTGAGGTTGCTGCTTTG
	Reverse	TTGACCCATACCGACCATGA
*CIT2*	Forward	TCGTTATATGGCTCAGCGTAAG
	Reverse	CCAGGTGCTACCTCGTATATTG
*ICL1*	Forward	GGTGGGACGCAATGTTCTAT
	Reverse	CTGTTGGAAGTCTGGGTAGTTAG
*MLS1*	Forward	GGCCAACTTGCCCACTATTA
	Reverse	CAAAGATGGAAGCGCTGATTG
*sPOX1*	Forward	ACACACTGGATGTGGACTCA
	Reverse	GTTTTCAACATGCAGCGCAA
*PCK1*	Forward	TGGTCTCAAGGTGAATCCAAATA
	Reverse	GGTGTGGCTCTGTCTTGATAA
*CAT2*	Forward	ACTTGGTTGATCTCCACATCTC
	Reverse	CAACATGTATGCCAGTCCAAAC
*SUC2*	Forward	TCCGTCTTTGCCGACTTATC
	Reverse	CTTAGAGTTACCACGGTCCAAA

### Inositol depletion

WT and *ino1*Δ cells were grown in I+ until the cells reached mid-log phase (A_550_ = 0.5–0.7). Cells were collected on a filter (0.45 μm, Millipore), washed twice with dH_2_O, and transferred to fresh medium with (I+, control) or without inositol (I−, inositol depletion in *ino1*Δ) for 4 h.

### Western blot analysis

Protein extracts were prepared by the boiling method (44). Extracts were resuspended in a loading buffer according to cell density (30 μl buffer/1.0 OD_550_), boiled at 100 °C for 5 min, and centrifuged at 10,000*g* for 5 min (44). Cleared supernatants (10 μl/lane) were loaded as parallel samples on two different 10% SDS-PAGE gels and electrotransferred to a polyvinylidene difluoride (PVDF) membrane (Millipore). The membranes were incubated with primary antibodies (1:500 anti-phospho (Thr-172)-AMPK (Cell Signaling Technologies); 1:1000 anti-polyhistidine antibody H1029 (Sigma) to detect endogenous Snf1; 1:50,000 anti-tubulin (Abcam) to normalize for protein loading in both membranes; 1;1000 appropriate secondary antibodies conjugated with europium), and fluorescence (Ex/Em 346/617 nm) was detected using a SpectraMax i3x multimode microplate reader with MiniMax 300 imaging cytometer and ScanLater Western blotting cartridge (Molecular Devices). Membranes were visualized with the SoftMax Pro software.

### ATP concentration

Cells were flash-frozen with liquid nitrogen. ATP levels were determined by the bioluminescence method described previously (82).

### Mitochondrial membrane potential

Cells were incubated with 500 nM tetramethylrhodamine (TMRM) for 30 min, washed once, and fluorescence was measured using flow cytometry (BD LSR II) at the Microscopy, Imaging & Cytometry Resources (MICR) Core at Wayne State University. Dye fluorescence is proportional to mitochondrial membrane potential and was calculated as the median fluorescence intensity (MFI) of single cells. Data presented are in arbitrary fluorescence units (AFUs).

### Glucose-induced proton efflux

Mid-log phase cells were obtained from 120 ml cultures. Cells were rapidly harvested by filtration (Millipore), washed four times with ice-cold water and stored on ice for 4 h to reduce metabolism to a minimum. Cells were resuspended in 10 ml of 250 mM sorbitol in a beaker at room temperature with rapid stirring in the presence of a pH electrode. After a stable reading was obtained, 100 mM glucose was added and the change in pH was followed until a constant value was achieved (15 min). Cells were collected on a preweighed filter. Dry weight of the cells was determined after incubating the cells overnight in an oven. The magnitude of the glucose-induced pH change is a measure of the ability of the H^+^-ATPase to pump protons (41, 56).

### Plasma membrane isolation and determination of Pma1 ATPase activity

Preparation of total extract and plasma membrane from yeast strain SF838-5Aα was done as described previously (83, 84). ATPase activity was determined using the coupled enzyme assay (57), and contributions of Pma1, the vacuolar ATPase, and mitochondrial F-type ATPase were assessed by preincubation of the membrane samples with 100 μM sodium vanadate, 200 nM concanamycin A, and 5 μM oligomycin, respectively. Vanadate-sensitive ATPase activity (from Pma1) represented 50–65% of the total ATPase activity. Membrane preparations were either preincubated with VPA (1 mM) or with buffer (control) on ice for 30 min, and the mixture was then added to the coupled enzyme assay to assess direct inhibition by VPA. To determine if VPA inhibits Pma1 activity through a posttranslational modification, cells were incubated with VPA (1 mM) or dH_2_O for 30 min and ATPase activity was assayed.

### Determination of plasma membrane levels of Pma1

Cells were incubated with VPA (1 mM) or dH_2_O for 30 min and plasma membrane levels of Pma1 were detected through indirect immunofluorescence as described previously (60).

### Mass spectrometry of glycolytic metabolite levels

Cell cultures (10 ml) were grown to the mid-log phase and treated with VPA (1 mM) or dH_2_O for 30 min. Cells were then quenched, and metabolites were extracted as described (85). Quantification of glycolytic metabolites was determined by mass spectrometry at the Karmanos Cancer Institute Pharmacology Core. The values represent the concentration of each metabolite (μM) normalized to cell density measured at A_550_.

### Cytosolic pH measurement

Cytosolic pH (pH_c_) was measured using yeast pHluorin, a pH-sensitive and ratiometric green fluorescent protein (67). Wild-type cells were transformed with the construct pPGK-pHluorin, in which the yeast pHluorin is under control of a phosphoglycerate kinase promoter, a generous gift from Dr Rajini Rao (Johns Hopkins University). Cells were grown to the mid-log phase in selective medium, then harvested by centrifugation, washed, and resuspended in 1 mM MES buffer containing 2% glucose. 1 mM VPA was added, and fluorescence intensity was measured at excitation wavelengths of 405 and 485 nm and emission wavelength of 508 nm. A calibration curve was constructed using 50 mM MES buffer (50 mM MES, 50 mM HEPES, 50 mM KCl, 50 mM NaCl, 0.2 M NH_4_ acetate, 10 mM NaN3, 10 mM 2-deoxyglucose) titrated to pH 6–8. Cells were incubated for 1 h at 30 °C in the presence of 75 μM monensin and 10 μM nigericin. A standard curve of fluorescence was generated and used to calculate the pH_c_ from the observed fluorescence ratios.

## Data availability

All data are contained within the article.

## Conflict of interest

The authors declare that they have no conflicts of interest with the contents of this article.
